# Clinical manifestations, complications, and outcomes of patients with COVID-19 in Sudan: a multicenter observational study

**DOI:** 10.1186/s41182-021-00382-4

**Published:** 2021-11-14

**Authors:** Elfatih A. Hasabo, Fatima A. Ayyad, Sara A. M. Alam Eldeen, Malaz K. Noureldaim, Tibyan A. Abdallah, Yousra T. Ahmed, Safaa Badi, Waleed K. Khalid, Hind A. Mirghani, Yossra A. Mohammed, Lubaba A. Alkhider, Saada A. Hamad, Amna M. Tagelseed, Ethar H. A. Elsheikh, Noon H. Khalid, Samia A. Mohammed, Sara O. Elmobark, Ola O. Ali, Duaa A. Mohammed Ali, Mohamed S. Muneer, Heitham Awadalla, Elfatih M. Malik

**Affiliations:** 1 Faculty of Medicine, University of Khartoum, ElQasr Avenue, Khartoum, Khartoum state 11111 Sudan; 2 School of Medicine, Ahfad University for Women, Khartoum, Sudan; 3 Faculty of Medicine, National Ribat University, Khartoum, Sudan; 4 Faculty of Medical Laboratory, University of Alzaiem Alazhari, Khartoum, Sudan; 5 Faculty of Pharmacy, Omdurman Islamic University, Khartoum, Sudan; 6 Sudan Medical Specialization Board, Khartoum, Sudan; 7 Federal Ministry of Health, Khartoum, Sudan; 8 Department of Community Medicine, Faculty of Medicine, University of Khartoum, Khartoum, Sudan

**Keywords:** COVID-19, Clinical manifestations, In-hospital outcomes, Complications, Sudan, Severity

## Abstract

**Background:**

Coronavirus disease 2019 (COVID-19) is a pandemic caused by a newly discovered coronavirus. Although clinical manifestations of COVID-19 are mainly pulmonary, some patients have other systemic manifestations. This study aimed to describe the clinical finding and outcomes in Sudanese patients diagnosed with COVID-19.

**Methods:**

This retrospective observational study is based on documented files that included patients diagnosed with COVID-19 in seven selected hospitals inside Khartoum. Clinical manifestations, complications and outcomes were extracted from patients’ records using an extraction form designed for this study.

**Results:**

Data of 243 patients diagnosed with COVID-19 were analyzed. The mean (SD) age in years was 55.8 (18.4). Out of 116 participants, 27 of them (23.3%) had severe disease, 15 (12.9%) were critically ill. 67.5% of patients were admitted to the hospital within 7 days from onset of symptoms; most of them were admitted to the wards (*n* = 140,72.5%). Fever (83.2%), cough (70.7%), and shortness of breath (69.2%) were the most commonly recorded clinical manifestations. Sepsis (9.8%) and acidosis (7.8%) were the most frequently reported complications. Death was the final outcome in 21.4% (56/243). Older age and presence of diabetes were found significantly associated with in-hospital death. The laboratory results showed high CRP in 85.6% (119/139), high ferritin in 88.9% (24/27), lactate dehydrogenase had a median of 409.0 (359–760), D-dimer had a median of 3.3 (1.2–16. 6), and 53/105 (50.5%) had low albumin.

**Conclusions:**

Fever was the most mentioned sign among the participants, followed by fatigue. Cough and shortness of breath were the most commonly recorded pulmonary symptoms manifested. Our study showed multiple variables were associated with in-hospital death. The mortality rate was high among severe and critically ill patients diagnosed with COVID-19.

## Introduction

COVID-19 is an infectious disease that rapidly evolved into a pandemic caused by a newly discovered coronavirus. Coronaviruses were identified for the first time in the mid-1960s [1], and the new coronavirus strain, namely severe acute respiratory syndrome coronavirus 2 (SARS-Cov-2), is related to the cluster of acute respiratory diseases in Wuhan, China, in December 2019 [2]. There were two highly infectious human coronaviruses in the past decades: the coronavirus responsible for the severe acute respiratory syndrome (SARS-Cov) and the coronavirus responsible for the Middle East respiratory syndrome (MERS-Cov) (1). They caused lower respiratory tract infections and extra-pulmonary manifestations, leading to an increase in the mortality rates of thousands of cases up to 50% in specific populations [2]. The COVID-19 symptoms and signs in most infected people will be mild-to-moderate respiratory diseases and recover with supportive treatment (2). Older people and those with comorbidities like cardiovascular disease, chronic respiratory disease, diabetes, cancer, and other immune diseases are more likely to develop a severe form of the disease [3].

Although clinical manifestations of COVID-19 are mainly pulmonary, some patients have other systemic manifestations such as cardiovascular, neurological, hematopoietic, and immune system manifestations [2]. Besides, several data reported that COVID-19 should be considered as a multisystem disease in which patients can present with chest tightness and palpitations instead of cough and other respiratory symptoms [2]. Furthermore, it was found that lymphopenia was the prevalent hematological findings in many patients [4]. Additionally, the high C-reactive protein (CRP), serum ferritin, lactate dehydrogenase (LDH), and several other immunological markers were associated with an increased risk of COVID-19 and death [5]. Also, coagulopathy has been reported in different cases, manifested by elevated D-dimer, prothrombin time (PT), and partial thromboplastin time (PTT) [6]. The risk of acute respiratory distress syndrome (ARDS) is associated with higher D-dimer and prothrombin time [7].

Several risk factors that increase mortalities among patients diagnosed with COVID-19 have been identified, such as older age, male sex, hypertension, diabetes, and cardiovascular diseases. In addition to that, there is still uncertainty regarding the patient of potential confounding [8].

On the 13th of March 2020, Sudan reported its first novel coronavirus case in Khartoum, and up to Monday 25th of January 2021, the total number of patients diagnosed with COVID-19 reached 28,522, including 1722 associated deaths, CFR: 6.5% [9]. Unfortunately, comprehensive knowledge of COVID-19 remains incomplete, and many essential features are still unknown. Moreover, since the emergence of COVID-19, the disease presenting symptoms were changing with time and place, and there were no previous published studies about outcomes of patients with COVID-19 in Sudan. We conducted this study to describe the clinical findings and outcomes in patients with COVID-19 in Sudan, Africa.

## Materials and methods

### Study design and population

This retrospective observational study included patients diagnosed with COVID-19 between the 1st of April and 30th of September 2020 in 7 selected hospitals inside Khartoum State, Sudan. Khartoum State is the capital of Sudan, and it has about 20 percent of the country’s total population, according to the latest estimate from the Integrated Food Security Phase Classification (IPC).

The Ministry of Health established three main isolation centers in Khartoum state, involving Khartoum teaching hospital, Jebra trauma center, and The Universal hospital. Additionally, many hospitals in public and private sectors have identified several beds for patients with COVID-19. Our study was conducted in seven hospitals in Khartoum state, which are:

1. Jebra Hospital for emergency and injuries is a governmental hospital located in Jebra, block 19, Khartoum city, with 88 beds for confirmed cases, in addition to an intensive care unit.
2. Khartoum Teaching Hospital is a public hospital existing in Isbitalia Street, Khartoum city, with 30 beds for patients with COVID-19, in addition to an Intensive care unit equipped with mechanical ventilators.
3. The Universal Hospital is an acute care tertiary facility, in Bahri city, Kober East Alwaha, with a bed capacity of 248 inpatient rooms divided into six floors.
4. Royal Care Hospital is a private, tertiary care hospital located in Mohamed Salah El-Din Street, Khartoum city, with 20 beds available for patients with COVID-19, in addition to an Intensive care unit.
5. Al Saha Specialized Hospital is a private hospital in Khartoum city, with a 20–30 bed capacity for patients with COVID-19.
6. Omdurman Teaching Hospital is a governmental hospital situated in Omdurman city, with 40 beds for patients with COVID-19.
7. Al-Baraha Medical City is a private hospital located et al.-Baraha Street, Bahri city, with 30 beds for patients with COVID-19.

We included Sudanese patients diagnosed with COVID-19 using nasopharyngeal swab tested by RT-PCR, 18 years old and older, and admitted to the mentioned hospitals. We excluded all pregnant women, asymptomatic patients, and patients lacking information necessary for the study in their files.

Patients were classified and treated according to the COVID-19 case management protocols released by the Federal Ministry of Health of the Republic of Sudan during the epidemic [10]. The mild form of the disease was defined as COVID-19 with uncomplicated, non-specific upper respiratory tract symptoms, for instance, sore throat, nasal congestion, and cough, in addition to mild general manifestations, including fever and malaise. The moderate disease was defined as uncomplicated pneumonia with oxygen saturation > 92% on room air and radiological evidence (typically bilateral ground-glass opacities). Patients with the severe disease are typically in respiratory distress with oxygen saturation < 92 on room air and radiological evidence of severe pneumonia. After assessing and classifying patients, those with moderate disease were admitted to isolation areas and treated empirically with amoxicillin 500 mg/8 h + azithromycin 500 mg daily for 3 days, with hydroxy-chloroquine 400 mg/12 h for one day followed by 400 mg for a total of 5 days. Patients with severe disease were admitted to a monitored bed with supplemental oxygen to keep saturation > 92%, and ceftriaxone 1 g/24 h + azithromycin 500 mg/24 h were prescribed, in addition to hydroxy-chloroquine 400 mg/12 h for one day, followed by 400 mg for a total of 5 days. Antivirals and immunomodulators were preserved for specific cases. Critically ill patients were admitted to ICUs and treated as severe sepsis. Non-invasive positive pressure ventilation/endotracheal intubation and mechanical ventilation were used to manage ARDS [10].

### Sampling technique and data collection

We covered all patients diagnosed with COVID-19 in our seven selected and previously mentioned hospitals, from the 1st of April to the 30th of September 2020. Data were collected from patients’ records and laboratory sheets using an extraction form. The form contained the following variables: sociodemographic characteristics, the severity of the disease, comorbidities, vital signs, clinical manifestations, duration from the onset of symptom to hospital admission, admission area, in-hospital complications, length of stay, short- and long-term in-hospital outcomes, and laboratory investigations including [red blood cells (RBCs), hemoglobin, hematocrit, white blood cells (WBCs), platelets, CRP, erythrocyte sedimentation rate (ESR), ferritin, lactate dehydrogenase, random blood sugar, sodium, potassium, blood urea nitrogen, creatinine, alanine aminotransferase, aspartate aminotransferase, alkaline phosphatase, total bilirubin, albumin and D-dimer].

### Statistical analysis plan

We described the data for all patients diagnosed with COVID-19 using SPSS version 25. Continuous data were presented as mean ± standard deviation (SD) or median (interquartile range). Categorical data were presented as numbers (percentages). Test of normality was used to find the distribution of continuous variables. Spearman’s correlation was used to find correlation of variables with severity of the disease or length of stay. A univariate logistic regression was used to find predictors of in-hospital death in patients with COVID-19. A *P*-value less than 0.05 is considered significant.

## Results

A total of 393 files were reached in this study, out of which 150 files were excluded due to incomplete necessary information (age, gender, comorbidities, presentation, complications, and the outcome). Accordingly, 243 files were included in this study.

### Sociodemographic data of patients with COVID-19

The mean age of the patients was 55.8 ± 18.4 years, and just over half of them (*n* = 126, 51.9%) were 60 years or above. More than half of the participants were males (*n* = 145, 59.7%) (Table 1).

**Table 1:** Sociodemographic characteristics and comorbidities of patients with COVID-19 and their association with the final outcome (*N* = 243)

Variables	*N* (%) or mean ± SD	Univariate logistic regression for predictors of in-hospital death			
		OR	Lower CI	Upper CI	P-value
Age, years, mean ± SD	55.8 ± 18.4	1.021	1.002	1.039	0.026
Age groups (years)					
18–29	34 (14.0)				
30–39	22 (9.1)				
40–49	24 (9.9)				
50–59	37 (15.2)				
60–70	69 (28.4)				
> 70	57 (23.5)				
Gender					
Male (reference)	145 (59.7)	–	–	–	–
Female	98 (40.3)	0.905	0.483	1.698	0.757
Comorbidities					
Hypertension	85 (35.0)	0.701	0.359	1.367	0.297
Diabetes	75 (30.9)	1.909	1.012	3.602	0.046
Cardiovascular disease	17 (7.0)	1.587	0.533	4.727	0.407
Asthma	16 (6.6)	0.23	0.03	1.784	0.16
Smoking	19 (7.8)	0.67	0.187	2.392	0.537
Chronic kidney disease	18 (7.4)	0.438	0.097	1.967	0.281
Cerebrovascular accident	9 (3.7)	3.1	0.802	11.988	0.101
Liver disease	1 (0.4)	–	–	–	1
Pulmonary tuberculosis	1 (0.4)	–	–	–	1
Alcohol	1 (0.4)	–	–	–	1

### Comorbidities

Hypertension and diabetes were the most commonly recorded comorbidities in (*n* = 85, 35%) and (*n* = 75, 30.9%) of patients, respectively (Table 1).

### In-hospital characteristics of patients with COVID-19

Respiratory manifestations were the most common clinical features among patients diagnosed with COVID-19 (*n* = 198, 81.5%), and a large proportion of them had a cough (*n* = 140, 70.7%). Fever was the leading complaint in the vast majority of patients (*n* = 144, 83.2%). Also, gastrointestinal features were presented in a small minority of patients (*n* = 42, 17.3%), and diarrhea was the most common feature (*n* = 18, 42.9%). Furthermore, only a mere 9% of patients complained of neurological symptoms (*n* = 23, 9.5%), and in the vast majority, a disturbing level of consciousness was the main complaint (*n* = 21, 91.3%) (Table 2).

**Table 2:** Clinical manifestations: symptoms, hospital disposition, complications and severity of patients diagnosed with COVID-19 and their association with the final outcome

Variables	*n*	Number (%) or median (IQR)	Univariate logistic regression for predictors of in-hospital death			
			OR	Lower CI	Upper CI	*P*-value
Duration from the onset of symptom to hospital admission, days	169	5 (3–10)	1.002	0.939	1.07	0.943
7 days and less		114 (67.5)				
8–14 days		44 (26.0)				
15–30 days		11 (6.5)				
More than 30 days		0 (0.0)				
Site of disposition at admission	193					
Ward deposition (reference)		140 (72.5)	–	–	–	–
HDU deposition^a^		50 (25.9)	5.231	2.538	10.781	**< 0.001**
ICU deposition^a^		3 (1.6)	2.833	0.246	32.666	0.404
Manifestations						
Pulmonary manifestations	198					
Cough		140 (70.7)	0.627	0.308	1.279	0.2
Shortness of breath		137 (69.2)	3.392	1.347	8.538	**0.010**
Hemoptysis		5 (2.5)	0.899	0.098	8.258	0.925
Chest pain		12 (6.1)	–	–	–	0.999
Sore throat		32 (16.2)	0.326	0.094	1.127	0.077
Congestion/runny nose		9 (4.5)	–	–	–	0.999
General manifestations	173					
Fever		144 (83.2)	1.263	0.445	3.588	0.661
Fatigue		58 (33.5)	0.887	0.4	1.967	0.796
Headache		40 (23.1)	0.159	0.036	0.697	**0.015**
Body aches		17 (9.8)	1.241	0.378	4.069	0.722
Loss of smell		4 (2.3)	–	–	–	0.999
Loss of taste		1 (0.6)	–	–	–	1
Neurological manifestations	23					
Disturbed level of consciousness		21 (91.3)	–	–	–	0.999
Hemiparesis		4 (17.4)	1.111	0.129	9.605	0.924
Aphasia		3 (13.0)	2.444	0.19	31.526	0.493
Hemi sensory deficit		0 (0.0)	–	–	–	–
Visual loss		0 (0.0)	–	–	–	–
Gastrointestinal manifestations	42					
Nausea/vomiting		15 (35.7)	2.933	0.711	12.108	0.137
Diarrhea		18 (42.9)	1.154	0.289	4.608	0.839
Abdominal pain		13 (30.9)	0.788	0.171	3.621	0.759
Loss of appetite		8 (19.0)	0.343	0.037	3.161	0.345
Vascular manifestation	3					
DVT^a^		2 (66.7)	–	–	–	1
Coronary artery disease		1 (33.3)	–	–	–	1
Bowel ischemia		0 (0.0)	–	–	–	–
Limb ischemia		0 (0.0)	–	–	–	–
In-hospital complications (yes)	243	70 (28.8)				
Sepsis		24 (9.8)	15.074	5.751	39.511	**< 0.001**
Acidosis		19 (7.8)	32.711	8.986	119.081	**< 0.001**
Respiratory failure		17 (6.9)	44.758	9.753	205.392	**< 0.001**
Acute kidney injury		15 (6.2)	21.647	5.800	80.791	**< 0.001**
Acute respiratory distress syndrome		12 (4.9)	6.533	1.971	21.661	** 0.002**
Hyponatremia		10 (4.1)	0.44	0.054	3.559	0.441
Alkalosis		8 (3.3)	7.480	1.718	32.558	** 0.007**
Hypokalemia		8 (3.3)	1.371	0.268	7.026	0.705
Shock		6 (2.5)	–	–	–	0.999
Hyperkalemia		5 (2.1)	–	–	–	0.999
Acute cardiac injury		4 (1.6)	4.205	0.576	30.677	0.157
Heart failure		2 (0.8	–	0		0.999
GIT bleeding^a^		2 (0.8)		0		0.999
Hypernatremia		1 (0.4)	–	–	–	1
Acute liver injury		0 (0.0)	–	–	–	–
Disseminated intravascular coagulation		0 (0.0)	–	–	–	–
Severity of the disease	116					
Mild (reference)		45 (38.8)	–	–	–	–
Moderate		29 (25.0)	3.259	0.282	37.687	0.344
Severe		27 (23.3)	40.857	4.899	340.728	** 0.001**
Critically ill		15 (12.9)	286.000	23.977	3411.477	**< 0.001**

Bold values mean these varibles were statistically significant

^a^HDU, high dependency unit; ICU, intensive care unit; DVT, deep vein thrombosis; GIT, gastrointestinal tract

Most of the patients reached the hospital within 7 days from the onset of symptoms (*n* = 114/169, 67.5%), and nearly half of them stayed at the hospital for 7 days or less (*n* = 98/201, 48.8%). A significant majority of the participants were admitted to wards (*n* = 140/193, 72.5%) (Table 2).

### Laboratory investigations

Regarding vital signs, patients had a median heart rate of 90 (81–103) beats per minute and median respiratory rate of 24 (20–36) breath per minute. Most of them were within the hypertensive systolic range (129/196, 65.8%) and hypertensive diastolic range (106/196, 54.1%) (Table 3).

**Table 3:** Vital signs and laboratory investigations among patients diagnosed with COVID-19 and their association with the final outcome

Variables	*n*	Number (%) or median (IQR)	Univariate logistic regression for predictors of in-hospital death			
			OR	Lower CI	Upper CI	*P*-value
Vital signs						
Heart rate, beat/min	189	90 (81–103)	1.052	1.030	1.076	**< 0.001**
Systolic BP^a^, mmHg	196	131 (118.3–145)	0.985	0.969	1.001	0.067
Normal		59 (30.1)				
Hypertension		129 (65.8)				
Hypertensive crisis		8 (4.1)				
Diastolic BP^a^, mmHg	196	80 (70.25–90)	1.001	0.999	1.002	0.47
Normal		85 (43.4)				
Hypertension		106 (54.1)				
Hypertensive crisis		5 (2.6)				
Respiratory rate, breath/min	116	24 (20–36)	1.061	1.022	1.103	**0.002**
Temperature, centigrade	93	37 (36.5–37.8)	0.956	0.776	1.178	0.674
SpO^2a^, percentage	182	95 (89–98)	0.903	0.866	0.942	**< 0.001**
Laboratory Investigation						
Red blood cells counts, million/mm^3^	128	4.3 (3.6–4.9)	0.566	0.345	0.928	**0.024**
Hemoglobin, g/dL	161	11.9 (10.1–13.6)	0.765	0.651	0.899	**0.001**
Hematocrit, percentage	129	36 (31–41)	0.894	0.836	0.956	**0.001**
WBCs counts^a^, × 103/μL	164	7.3 (5,2–12.1)	1.144	1.074	1.218	**< 0.001**
Leukopenia	164	15 (9.1)				
Normal		102 (62.2)				
Leukocytosis		47 (28.7)				
Monocyte, × 10^3^/μL	103	0.5 (0.4–0.8)	1.624	0.854	3.087	0.139
Lymphocyte, × 10^3^/μL	145	1.3 (0.8–2.0)	1.014	0.776	1.326	0.919
Lymphopenia	145	85 (58.6)				
Normal		59 (40.7)				
Lymphocytosis		1 (0.7)				
Neutrophils, × 10^3^/μL	168	5.25 (3.19–9.8)	1.125	1.049	1.205	**0.001**
Neutropenia	168	11 (8.0)				
Normal		79 (57.2)				
Neutrophilia		48 (34.8)				
Platelets, × 10^9^/L	87	247.5 (187.5–342.3)	0.997	0.994	1.001	0.101
CRP, mg/dl^a^	112	62 (18.1–143.8)	1.006	1.002	1.010	**0.003**
Normal CRP level		14 (12.5)				
High CRP level		98 (87.5)				
ESR, mm/hour	64	40 (15–68)	1.018	0.998	1.039	0.078
Ferritin, ng/ml	22	651.9 (470.4–1472.8)	1	0.999	1.001	0.658
Normal Ferritin level		1 (4.5)				
High Ferritin level		21 (95.5)				
Lactate dehydrogenase, unit/liter	19	409.0 (359–760)	1	0.998	1.001	0.688
Random blood sugar, mg/dL	112	141 (102.5–261.8)	1.004	1.001	1.008	**0.024**
Sodium, mmol/L	144	135 (130–139)	1.04	0.994	1.088	0.086
Potassium, mmol/L	146	3.8 (3.5–4.3)	1.742	1.093	2.777	**0.020**
Blood Urea nitrogen, mg/dL	149	30 (17–50.5)	1.010	1.004	1.018	**0.003**
Creatinine, mg/dL	151	1 (0.7–2.2)	1.003	0.992	1.014	0.585
Alanine aminotransferase, unit /liter	78	26 (19–43.7)	1.014	1.002	1.025	**0.022**
Aspartate aminotransferase, unit /liter	73	28 (17–56.7)	1.018	1.002	1.035	**0.024**
Alkaline phosphatase, unit /liter	70	96 (63.5–119)	1.001	0.998	1.003	0.55
Total bilirubin, mg/dl	55	0.6 (0.3–0.9)	3.426	1.087	10.803	**0.036**
Albumin, g/dl	88	3.5 (2.9–4.2)	1.016	0.985	1.048	0.312
Low Albumin level		44 (50.0)				
Normal Albumin level		41 (46.6)				
High Albumin level		3 (3.4)				
D-dimer	26	3.3 (1.2–16. 6)	0.939	0.804	1.097	0.429

Bold values mean these varibles were statistically significant

^a^BP, blood pressure; SpO^2^, saturation of peripheral oxygen; WBCs, white blood cells; CRP, C-reactive protein; ESR, estimation sedimentation rate

Regarding laboratory tests, though most of patients had normal WBCs and neutrophil counts (102/164, 62.2%) and (79/168, 57.2%), respectively, most of them had lymphopenia (85/145, 58.6%).  Other important findings include elevated CRP (98/112, 87.5%), high ferritin level (21/22, 95.5%) and hypoalbuminemia (44/88, 50%) (Table 3).

### In-hospital complications, severity, interventions, and outcomes of COVID-19

#### Complications

Although, three-quarters of patients did not develop any complications (*n* = 173/243, 71.2%), sepsis (*n* = 24/243, 9.8%) and acidosis (*n* = 19/243, 7.8%) were the most commonly mentioned complications (Table 2).

### Severity of the disease

Out of 243 patients enrolled, only 116 were classified in the documents according to the disease severity score. 38% of those patients had the mild disease (*n* = 45, 38.8%), and precisely, a quarter of them had moderate disease (29, 25.0%). Additionally, severe disease was found in (*n* = 27, 23.3%), and a few of the patients were critically ill (15, 12.9%) (Table 2).

### Interventions

Concerning the need for respiratory support, nearly a fifth of the participants (*n* = 47/182, 25.8%) needed this intervention, in which more than half of them required non-invasive ventilation (*n* = 25, 53.2%), compared to (*n* = 13, 27.7%) who needed invasive ventilation. In the remaining files (*n* = 9, 19.1%), the type of ventilation was not recorded (Table 4).

**Table 4:** Respiratory support and outcomes of patients diagnosed with COVID-19 and the association of respiratory support with final outcome

Variables	*n*	Number (%) or median (IQR)	Univariate logistic regression			
			OR	Lower CI	Upper CI	*P*-value
Respiratory support	182					
Not used		135 (74.2)	–	–	–	–
Used		47 (25.8)	16.612	7.113	38.800	**< 0.001**
Type of respiratory support used	38					
Non-invasive		25 (65.8)				
Invasive		13 (34.2)				
Short term outcome (after 15 days)	193					
Discharge home		131 (67.9)				
Death		34 (17.6)				
HDU transfer^a^		5 (2.6)				
ICU transfer^a^		19 (9.8)				
Others		4 (2.1)				
Outcome	243					
Discharge home		191 (78.6)				
Death		52 (21.4)				
In-hospital stay from admission to the outcome, Days	201	8 (4–14.5)				
7 days and less		98 (48.8)				
8–14 days		53 (26.4)				
15–30		42 (20.9)				
More than 30		8 (3.9)				

Bold values mean these varibles were statistically significant

^a^HDU, High Dependency Unit; ICU, Intensive Care Unit

### Outcomes

*n* = 131/193, 67.9% of participants were discharged home within 15 days of their admission and *n* = 191/243, 78.6% discharged in the long-term (Table 4).

### Factors influencing the outcome in patients with COVID-19

In univariate logistic regression, some factors were found significantly associated and predict the final outcome (in-hospital death). These factors were: increase in age (OR: 1.021, CI 95% 1.002–1.039; *P* = 0.026), presence of diabetes (OR: 1.909, CI 95% 1.012–3.602; *P* = 0.046), deposition to HDU at the time of admission (OR: 5.231, CI 95% 2.538–10.781; *P* ˂ 0.001), admission with shortness of breath (OR: 3.392, CI 95% 1.347–8.538; *P* = 0.010), absence of headache at the time of admission (OR: 0.159, CI 95% 0.036–0.697; *P* = 0.015), sepsis (OR: 15.074, CI 95% 5.751–39.511; *P* ˂ 0.001), acidosis (OR: 32.711, CI 95% 8.986–119.081; *P* ˂ 0.001), respiratory failure (OR: 44.758, CI 95% 9.753–205.392; *P* ˂ 0.001), acute kidney injury (OR: 21.647, CI 95% 5.800–80.791; *P* ˂ 0.001), acute respiratory distress syndrome (OR: 6.533, CI 95% 1.971–21.661; *P* = 0.002), alkalosis (OR: 7.480, CI 95% 1.718–32.558; *P* = 0.007), patient with severe disease (OR: 40.857, CI 95% 4.899–340.728; *P* = 0.001) and patient with critically ill disease (OR: 286.000, CI 95% 23.977–3411.477; *P* ˂ 0.001). The predictors of in-hospital death in laboratory investigations and vital signs were: increase in heart rate (OR: 1.052, CI 95% 1.030–1.076; *P* ˂ 0.001), increase in respiratory rate (OR: 1.061, CI 95% 1.022–1.103; *P* = 0.002), decrease in SpO^2^ (OR: 0.903, CI 95% 0.866–0.942; *P* ˂ 0.001), low RBCs counts (OR: 0.903, CI 95% 0.866–0.942; *P* = 0.024), low hemoglobin concentration (OR: 0.765, CI 95% 0.651–0.899; P = 0.001), low hematocrit (OR: 0.894, CI 95% 0.836–0.956; *P* = 0.001), high WBCs (OR: 1.144, CI 95% 1.074–1.218; *P* ˂ 0.001), high neutrophils (OR: 1.125, CI 95% 1.049–1.205; *P* = 0.001), high CRP (OR: 1.006, CI 95% 1.002–1.010; *P* = 0.003), high random blood sugar (OR: 1.004, CI 95% 1.001–1.008; *P* = 0.024), high potassium (OR: 1.742, CI 95% 1.093–2.777; *P* = 0.020), high blood urea nitrogen (OR: 1.010, CI 95% 1.004–1.018; *P* = 0.003), high alanine aminotransferase (OR: 1.014, CI 95% 1.002–1.025; *P* = 0.022), high aspartate aminotransferase (OR: 1.018, CI 95% 1.002–1.035; *P* = 0.024), high total bilirubin (OR: 3.426, CI 95% 1.087–10.803; *P* = 0.036) and usage of respiratory support (OR: 16.612, CI 95% 7.113–38.800; *P* ˂ 0.001) (Tables 1, 2, 3, 4).

### Factors associated with COVID-19 severity

Several factors were found to be significantly positively correlated with the disease severity. These factors includes: Age (*p*-value ≤ 0.001, *R* = 0.497), diabetes (*p*-value = 0.016, *R* = 0.224), hypertension (*p*-value ≤ 0.001, *R* = 0.373), cardiovascular disease (*p*-value = 0.002, *R* = 0.288), cerebrovascular disease (*p*-value = 0.005, *R* = 0.258), shortness of breath (*p*-value ≤ 0.001, *R* = 0.479), acute respiratory distress syndrome (*p*-value = 0.015, *R* = 0.233), respiratory failure (*p*-value ≤ 0.001, *R* = 0.352), sepsis (*p*-value = 0.002, *R* = 0.292), acidosis (*p*-value ≤ 0.001, *R* = 0.400), alkalosis (*p*-value = 0.002, *R* = 0.292), acute kidney injury (*p*-value = 0.017, *R* = 0.230), hypokalemia (*p*-value = 0.025, *R* = 0.215), shock (*p*-value = 0.031, *R* = 0.208), heart rate (*p*-value = 0.003, *R* = 0.276), respiratory rate (*p*-value < 0.001, *R* = 0.544), WBCs count (*p*-value ≤ 0.001, *R* = 0.551), neutrophil counts (*p*-value ≤ 0.001, *R* = 0.651), CRP level (*p*-value ≤ 0.001, *R* = 0.718), ESR level (*p*-value = 0.004, *R* = 0.419), random blood sugar (*p*-value ≤ 0.001, *R* = 0.441), Sodium (*p*-value = 0.005, *R* = 0.321), blood Urea level (*p*-value ≤ 0.001, *R* = 0.435), Creatinine level (*p*-value = 0.006, *R* = 0.293), alanine aminotransferase level (*p*-value = 0.002, *R* = 0.427), aspartate aminotransferase level (*p*-value ≤ 0.001, *R* = 0.650), alkaline aminotransferase (*p*-value ≤ 0.001, *R* = 0.588), and total bilirubin level (*p*-value = 0.047, *R* = 0.349).

While on the other hand factors that were found to be significantly negatively correlated with the disease severity includes: smoking (*p*-value ≤ 0.001, *R*−0.325), headache (*p*-value ≤ 0.001, *R* = −0.483), body aches (*p*-value = 0.025, *R* = −0.237), sore throat (*p*-value ≤ 0.001, *R* = −0.357), runny nose (*p*-value = 0.027, *R* = −0.222), using respiratory support (*p*-value ≤ 0.001, *R* = −0.589), diastolic blood pressure (*p*-value ≤ 0.001, *R* =  − 0.354), SaPO2 (*p*-value ≤ 0.001, *R* = −0.730), lymphocyte counts (*p*-value ≤ 0.001, *R* = −0.439), RBCs counts (*p*-value = −0.511), hemoglobin level (*p*-value ≤ 0.001, *R* = −0.496), hematocrit level (*p*-value ≤ 0.001, *R* = −0.542), and D-dimer (*p*-value ≤ 0.001, *R* = −0.826) (Tables 4, 5, 6, 7).

**Table 5:** Correlation between participant’s characteristics and comorbidities with length of stay and severity of the disease

Variables	Correlation			
	Length of in-hospital stay		Severity of the disease	
	*R*	*P*-value	*R*	*P*-value
Age	−0.012	0.866	0.497	** < 0.001**
Gender	−0.039	0.581	−0.078	0.407
Comorbidities				
Diabetes	−0.052	0.46	0.224	**0.016**
Hypertension	0.059	0.407	0.373	** < 0.001**
Asthma	−0.083	0.243	−0.069	0.485
Pulmonary TB	0.036	0.612	0.004	0.963
Cardiovascular disease	0.081	0.254	0.288	**0.002**
Chronic kidney disease	−0.071	0.317	0.147	0.109
Chronic liver disease	0.002	0.979	0.147	0.115
Cerebrovascular disease	−0.142	0.044	0.258	**0.005**
Smoking	−0.09	0.206	−0.325	** < 0.001**
Alcohol	−0.035	0.618	–	–

Bold values mean these varibles were statistically significant

**Table 6:** Correlation between clinical manifestations and complications with length of stay and severity of the disease

Variables	Correlation			
	Length of in-hospital stay		Severity of the disease	
	*R*	*P*-value	*R*	*P*-value
Duration from Onset of symptom to admission	0.015	0.859	0.187	0.071
General manifestations				
Fever	0.066	0.353	0.078	0.469
Headache	−0.058	0.411	−0.483	** < 0.001**
Fatigue	0.01	0.891	−0.067	0.53
Loss of the smell sensation	0.032	0.656	−0.127	0.234
Loss of taste	−0.02	0.777	−0.127	0.234
Body aches	−0.003	0.968	−0.237	0.025
Neurological manifestations				
Disturbed level of consciousness	−0.145	0.041	−0.166	0.647
Hemiparesis	−0.008	0.913	0.248	0.489
Aphasia	−0.076	0.285	−0.58	0.079
Pulmonary manifestations				
Cough	−0.115	0.105	0.107	0.29
Shortness of breath	0.171	**0.015**	0.479	** < 0.001**
Sore throat	−0.159	0.044	−0.357	** < 0.001**
Runny nose	0.051	0.471	−0.222	0.027
Chest Pain	0.107	0.129	−0.034	0.753
Hemoptysis	0.032	0.652	0.135	0.182
Gastrointestinal manifestations				
Nausea and vomiting	−0.035	0.626	0.325	0.13
Diarrhea	−0.059	0.407	−0.095	0.665
Abdominal pain	0.02	0.778	−0.029	0.897
Loss of appetite	−0.69	0.69	−0.02	0.927
Vascular manifestations				
DVT	−0.109	0.125	−0.5	0.667
Coronary artery disease	−0.119	0.092	0.5	0.667
In-hospital complications				
Acute respiratory distress syndrome	0.165	**0.019**	0.233	**0.015**
Respiratory failure	−0.136	0.054	0.352	** < 0.001**
Acute cardiac injury	0.015	0.829	–	–
Heart failure	−0.024	0.737	0.153	0.113
Sepsis	0.049	0.492	0.292	**0.002**
Acidosis	−0.192	**0.006**	0.4	** < 0.001**
Alkalosis	0.065	0.357	0.292	**0.002**
Acute kidney injury	−0.007	0.924	0.23	**0.017**
Hyperkalemia	−0.032	0.649	0.153	0.113
Hypokalemia	−0.043	0.548	0.215	**0.025**
Hypernatremia	0.092	0.193	–	**–**
Hyponatremia	−0.076	0.258	−0.036	0.715
Shock	−0.075	0.292	0.208	**0.031**
Gastrointestinal bleeding	−0.035	0.618	0.153	0.113
Disease severity	−0.02	0.841	–	–
Respiratory support	−0.136	0.086	−0.589	** < 0.001**

Bold values mean these varibles were statistically significant

**Table 7:** This table shows the association between vital signs and laboratory investigations and length of stay and severity of the disease

Variables	Correlation			
	Length of in-hospital stay		Severity of the disease	
	*R*	*P*-value	*R*	*P*-value
Heart rate	−0.078	0.323	0.276	**0.003**
Systolic blood pressure	0.114	0.141	−0.034	0.723
Diastolic blood pressure	−0.048	0.539	−0.354	** < 0.001**
Respiratory rate	0.056	0.5789	0.544	** < 0.001**
Temperature	−0.033	0.765	0.135	0.332
SpO^2^	−0.063	0.429	−0.73	** < 0.001**
WBCs counts	−0.08	0.344	0.551	** < 0.001**
Monocyte count	−0.038	0.72	0.192	0.128
Lymphocyte count	0.001	0.988	−0.439	** < 0.001**
Neutrophil count	−0.029	0.755	0.651	** < 0.001**
Red blood cells counts	−0.091	0.346	−0.511	** < 0.001**
Haemoglobin level	−0.03	0.72	−0.496	** < 0.001**
Hematocrit	−0.118	0.215	−0.542	** < 0.001**
Platelet counts	0.103	0.23	−0.177	0.1
CRP level	−0.01	0.922	0.718	** < 0.001**
ESR level	0.127	0.34	0.419	**0.004**
Ferritin	0.251	0.301	0	1
Lactate dehydrogenase	0.263	0.292	0.316	0.604
Random blood sugar	−0.073	0.472	0.441	** < 0.001**
Sodium	0.252	**0.005**	0.321	**0.005**
Potassium	−0.041	0.651	0.042	0.72
Blood urea	0.026	0.765	0.435	** < 0.001**
Creatinine	0.132	0.131	0.293	**0.006**
Alanine aminotransferase	−0.04	0.74	0.427	**0.002**
aspartate aminotransferase	−0.043	0.73	0.65	** < 0.001**
Alkaline aminotransferase	0.232	0.063	0.588	** < 0.001**
Total bilirubin	0.105	0.471	0.349	**0.047**
D-dimer	0.405	0.056	−0.826	** < 0.001**

Bold values mean these varibles were statistically significant

### Factors associated with length of in-hospital stay

Several factors were found to be significantly correlated with the length of in-hospital stay, these factors are: cerebrovascular disease (*p*-value 0.044, *R* = −0.142), disturbed level of consciousness (*p*-value = 0.041, *R* = −0.145), shortness of breath (*p*-value = 0.015, *R* = 0.171), shortness of breath (*p*-value = 0.044, *R* = −0.159), acute respiratory distress syndrome (*p*-value = 0.019, *R* = 0.165) and Acidosis (*p*-value 0.006, *R* = −0.192) (Tables 4, 5, 6, 7).

## Discussion

This study is an observational, retrospective study to describe the clinical characteristics, complications, and outcomes of patients with COVID-19, conducted in the isolation centers of Khartoum Sudan, in April 2020, with 243 patients enrolled. We found that men are more susceptible to SARS-COV-2 (59.7%) than women (40.3%), which is similar to other studies conducted in Wuhan, China (73%) [11] and New York City (60%) [12], this may be due to several factors, including genetic factors such as the high expression of coronavirus receptors (ACE2) in men [13, 13, 14] or behavioral characteristics. A study conducted in Spain revealed that women have a greater sense of responsibility towards the COVID-19 pandemic than men [15].

Our results revealed that the most vulnerable age groups are the elderly, especially those aged between 60–70 years (28.4%), followed by the age group above 70 years (23.5%) as a result of weak immune functions [16].

In concrete with other studies [12, 17], our study revealed that hypertension is the most common underlying comorbidity among the studied cases (35%). At the same time, diabetes mellitus, cardiovascular diseases, and asthma were reported in 30.9%, 7%, and 6.6% of patients, respectively, and no HIV seropositive patients were identified. Moreover, we found that hypertension is the most chronic disease that can increase the severity of COVID19, with ACE2 becomes the likely explanation [18], followed by cerebrovascular and cardiovascular diseases.

When it comes to laboratory tests, leukocytosis, neutrophilia, and lymphopenia were found in (28.7%) (34.8%) and (58.6%) of participants, respectively. This result is consistent with other studies conducted in China [19] and United States [20]. Also, elevated CRP and hypoalbuminemia were identified in 87.5% and 50% of patients. Interestingly, we noticed a positive correlation between these parameters and the severity of COVID 19; using them may help to identify patients with severe disease and their need for hospitalization. [(*p* = 0.001) (*p* ≤ 0.0001), respectively].

The most common pulmonary symptoms were cough (70.7%), followed by shortness of breath (69%), sore throat (16.2%), and hemoptysis (2.5%). The development of these symptoms can be explained by the presence of severe pneumonia in patients with COVID-19. Furthermore, the severity of the disease at the time of presentation may result in such variation of the symptoms [21–23].

Although COVID-19 primarily presents with pulmonary manifestations, extra-pulmonary features have been mentioned with varying degrees of severity and frequency. General manifestations including fever and fatigue were reported in (83.2%) and (33.5%) of the cases. Our study identified that gastrointestinal symptoms were presented in (17.3%) of the patients, and diarrhea was the most commonly mentioned symptom, found in 42.9% of the participants, followed by nausea and vomiting (35.7%) and abdominal pain (30.9%). Similarly, Ramachal et al. reported diarrhea as the most common GI manifestation (14.7%), followed by nausea and vomiting (10.7%) and abdominal pain (2%) [24]. Also, Assiri et al. and Dawei et al. showed that diarrhea was the predominant GI symptom (22% and 10.1%), respectively [25, 26].

Additionally, neurological manifestations have been reviewed in this study, ranging from mild non-specific neurological symptoms to more severe ones. Headache was the most common neurological manifestation (32.1%). These findings were consistent with the previous study conducted in China (7). No documented CT brain reports were found in the examined files.

In terms of vital signs, the median for heart rate was 90 beats/min (*n* = 189), respiratory rate 24 breaths/min (*n* = 116), temperature 37 (*n* = 39) and SpO^2^ 95% (*n* = 182). The median systolic and diastolic blood pressures were 131 and 80, respectively. Of 243 patients, about 65.8% had high systolic blood pressure compared to 30.1% with normal blood pressure.

The majority of patients (75%) did not show any complications during the course of the disease. However, among the 243 patients, the most common complication was sepsis (9.9%), followed by acidosis (7.8%), respiratory failure (6.9%), AKI (6.2%), and ARDS (4.1%). With regard to the current study, sepsis and acidosis were the most commonly mentioned complications. In contrast, Chen et al. showed that ARDS and respiratory failure were the most common complications [19]. Therefore, early identification and proper treatment of critical cases are of high importance. As Sudan is a low-income country with a weak health system and poverty, patients cannot afford investigations such as blood cultures to identify organisms causing sepsis; hence, health care workers depend only on the patient’s clinical situation for assessment and choosing the proper antibiotics.

Among all reported symptoms, shortness of breath (SOB) and cough were the most prevalent symptoms among moderate and severe groups. Shortness of breath was reported in 137/198 (69.2%) of the studied patients, with a significant difference among patients, being most prevalent among severe ones (*p* ≤ 0.00001), indicating that SOB has a predictive value on disease severity. These findings are similar to a study conducted in China [27]. Headache and sore throat were documented in (23.1% and 16.2%) and are associated with mild and moderate COVID-19. (*p* ≤ 0.00001) (*p* ≤ 0.011), respectively.

Regarding the outcome, most admitted patients stayed for 7 days or less, 9.8% of patients required ICU admission. Also, 131 (67.9%) patients were discharged home compared to 34 (17.6%) who died. The mortality is considered of a high rate, and this can be explained by several factors, the most important of which is the worsening economic situation of the country, as this hinders the expansion of diagnosis, tracing the cases and contacts, doing investigations, and proper management of the cases. Another factor is drug insecurity, either unavailability of essential drugs or unaffordability of the price by the patients [28]. It is worth mentioning that low medical resources, shortage of personal protective equipment for HCWs, and deteriorating healthcare system directly influence patients’ care and survival. Furthermore, lack of commitment with precautions in the community like wearing masks and social distance, and late presentation of the patients to hospitals have a role in this alarming rate.

This study is considered the first multicenter observational study to describe the clinical manifestations, laboratory investigations, and outcomes for patients with COVID-19 in Sudan and one of the fewest studies in Africa. Additionally, it gives a detailed description and new information for all general, neurological, gastrointestinal, pulmonary, and vascular symptoms and investigations for patients with COVID-19.

However, our study had several limitations. First, it is a retrospective study based on documented files; therefore, although we collected many files, we excluded more than 200 files due to incomplete necessary information. We recommend that documentation practices among health care providers must be evaluated on an ongoing basis using audits. Also, an electronic entry system should be established. Second, we excluded all patients diagnosed and treated as COVID-19 based on their radiological findings only without RT-PCR in order to obtain more accurate and precise results, but we found that laboratory testing was not available for many patients, most probably due to financial reasons; this contributed to further losses in the numbers of patients. Third, we undoubtedly lost patients who had mild symptoms, diagnosed and treated in an outpatient setting.

## Conclusion

Among the general manifestations for patients diagnosed with COVID-19, fever was the most mentioned sign, followed by fatigue. Shortness of breath and cough were the most commonly recorded pulmonary symptoms manifested. Most of the patients did not show any in-hospital complications. Several factors were found associated with in-hospital death. The mortality rate was high, and it increased in severe and critically ill patients diagnosed with COVID-19.

## Data Availability

The datasets used and analyzed during this study are available from the corresponding author on reasonable request.

## Abbreviations

ACE: Angiotensin-converting enzyme 2
AKI: Acute kidney injury
ARDS: Acute respiratory distress syndrome
CFR: Case fatality risk/ratio
COVID-19: Coronavirus disease 2019
CRP: C-reactive protein
CVD: Cardiovascular diseases
ESR: Erythrocyte sedimentation rate
GI: Gastrointestinal
HDU: High dependency unit
ICU: Intensive care unit
IPC: Integrated Food Security Phase Classification
LDH: Lactate dehydrogenase
MERS-Cov: Middle east respiratory syndrome coronavirus
PT: Prothrombin time
PTT: Partial thromboplastin time
RBC: Red blood cell
RT-PCR: Reverse transcription-polymerase chain reaction
SARS-Cov-2: Severe acute respiratory syndrome coronavirus 2
SD: Standard deviation
SOB: Shortness of breath
SpO2: Saturation of peripheral oxygen
SPSS: Statistical Package for the Social Sciences
